# Molecular Detection of *Avipoxvirus* in Wild Birds in Central Italy

**DOI:** 10.3390/ani12030338

**Published:** 2022-01-29

**Authors:** Fabrizio Bertelloni, Renato Ceccherelli, Margherita Marzoni, Alessandro Poli, Valentina Virginia Ebani

**Affiliations:** 1 Department of Veterinary Sciences, University of Pisa, Viale delle Piagge 2, 56124 Pisa, Italy; fabrizio.bertelloni@unipi.it (F.B.); margherita.marzoni@unipi.it (M.M.); alessandro.poli@unipi.it (A.P.); 2 CRUMA-LIPU, Via delle Sorgenti 430, 57121 Livorno, Italy; apusvet.cruma@libero.it; 3 Centre for Climate Change Impact, University of Pisa, Via del Borghetto 80, 56124 Pisa, Italy

**Keywords:** *Avipoxvirus*, wild birds, aquatic birds, *Anseriformes*, PCR

## Abstract

**Simple Summary:**

*Avipoxviruses* (APVs) are responsible for diseases in domestic and wild birds. Currently, the disease in domestic animals is under control in many Countries by biosafety and vaccination. In wild birds, small disease events are frequently reported worldwide, but large outbreaks are generally rare. Nevertheless, some aspects of the epidemiology of these viruses are still unclear. In this study, we explored, through molecular investigations, the diffusion of APVs among wild birds, of different orders and species, without typical macroscopic lesions. A high percentage (43.33%) of positive specimens was detected, suggesting high diffusion of the viruses and a possible role of avian wildlife as a reservoir. Aquatic birds, mainly *Anseriformes*, were more often infected, probably in relation to the environment where they live; in fact, APVs are frequently transmitted by mosquitos, particularly abundant in humid areas.

**Abstract:**

*Avipoxviruses* (APVs) are important pathogens of both domestic and wild birds. The associated disease is characterized by skin proliferative lesions in the cutaneous form or by lesions of the first digestive and respiratory tracts in the diphtheritic form. Previous studies investigated these infections in symptomatic wild birds worldwide, including Italy, but data about the circulation of APVs in healthy avian wildlife are not available. The present study tested spleen samples from 300 wild birds without typical lesions to detect *Avipoxvirus* DNA. Overall, 43.33% of the samples scored positive. Aquatic birds were more frequently infected (55.42%) than other animals (26.40%), and in *Anseriformes,* high positivity was found (52.87%). The obtained results suggest that wild birds could be asymptomatic carriers of *Avipoxviruses*, opening new possible epidemiological scenarios.

## 1. Introduction

Members of the *Avipoxvirus* (APV) genus are the causative agents of serious diseases of wild and domestic birds worldwide [1]. Infected animals can develop a cutaneous or dry form and/or a diphtheritic or wet form. The first one is the most common condition, often caused by arthropod transmission of the virus; it is characterized by typical cutaneous papules that evolve in crusty scabs, mainly on unfeathered areas, such as comb, wattles, around beak and eyes, and legs. The second form, more frequent in commercial poultry flocks, is caused by inhalation or ingestion of the virus. In this form, the virus causes lesions on the mucous membranes of the mouth, pharynx, larynx, and sometimes the trachea [2,3].

Nowadays, 12 species are recognized and included in the *Avipoxvirus* genus by the International Committee on Taxonomy of Viruses (ICTV), but other species probably exist [4,5]. Virus species’ name originates from the typical host for which the virus exhibits the highest virulence. The most known APV are fowl poxvirus (FWPV), turkey poxvirus (TPW), pigeon poxvirus (PGPV), and canary poxvirus (CNPV) [6]. However, these agents are not strictly species-specific, and they may infect other avian species, usually exhibiting a lower virulence. Recently, molecular taxonomic investigations based on the locus *fpv140* divided APVs into five clades and different subclades, including the various species identified [6,7,8].

APV infections have been reported in more than 370 avian species from 23 orders, including domestic and wild birds [3,4,9]. Passeriformes have been found to be frequently infected by APV, but the infection has been often observed in Anseriformes, Galliformes, and Psittaciformes too [9].

Even though APV infections are well known, some aspects regarding their epidemiology are still unclear. The role of wild birds, mainly those that are asymptomatic, has not been completely investigated.

APV infections have been observed in wild birds with typical cutaneous lesions. In Italy, the etiologic virus was found in stone curlew (*Burhinus oedicnemus*) [10], common buzzard (*Buteo buteo*) [11,12], Japanese quail (Coturnix japonica), grey partridge (Perdix perdix), canary (Serinus canarius), song thrush (Turdus philomelos), pheasant (Phasianus colchicus), feral pigeon (Columba livia), gyrfalcon (Hierofalco rusticolus), hooded crow (Corvus corone), dunnock (Prunella modularis) [13], and griffon vultures (*Gyps fulvus*) [14].

However, to the best of our knowledge, surveys on the circulation of APVs in the asymptomatic wild avian population in Italy have not been carried out.

The aim of the present molecular investigation was to verify the spreading of *Avipoxvirus* among apparently asymptomatic wild birds that belong to different orders and species sampled in Central Italy.

## 2. Materials and Methods

### 2.1. Samples

From January 2017 to December 2019, spleen samples were collected from wild birds of different orders and species. The group of animals included birds hunted during regular hunting seasons in Tuscany, Italy. Gastrointestinal tracts, with the spleen and liver, were collected from each bird by hunters during domestic evisceration and placed in disposable sterile plastic bags. The head and legs of each animal were also collected to successively verify the presence of macroscopic lesions characteristic of *Avipoxvirus* infections. For each examined bird, species and gender were annotated. All samples were sent to the Laboratory of Avian Pathology of the Department of Veterinary Science, University of Pisa, where all the anatomic parts were macroscopically examined; each bird’s spleen was collected and stored separately at −20 °C for further analyses.

The second group of investigated animals included birds from a recovery center located in Tuscany, where they died; carcasses were sent to the Laboratory of Avian Pathology of the Department of Veterinary Science, University of Pisa, where they were submitted to necropsy. Each bird’s spleen was collected and stored at −20 °C for further analyses. For each examined bird, species and gender were annotated.

No animals were specifically sacrificed for this study.

### 2.2. Molecular Investigations

DNA was extracted from each spleen specimen using a commercial kit, the Tissue Genomic DNA Extraction Kit (Fisher Molecular Biology, Trevose, PA, USA), following the manufacturer’s instructions. The primers APV4bF (CAGCAGGTGCTAAACAACAA) and APV4bR (CGGTAGCTTAACGCCGAATA), targeting a 578 bp segment of *4b core protein* gene, were employed to detect APV DNA. The primer pair was previously described on the basis of the published *4b core protein* gene (P4b) sequence of fowl poxvirus strain HP444 and common to the APV genus [15,16].

PCR was performed in a final volume of 25 µL containing 12.5 μL of EconoTaq PLUS 2× Master Mix (Lucigen Corporation, Middleton, WI, USA), 0.5 μM of each primer, 3 μL of DNA, and distilled water to reach the final volume. Amplifications were performed in an automated thermal cycler (Gene-Amp PCR System 2700, Perkin-Elmer, Norwalk, CT, USA), applying the following conditions: initial denaturation at 95 °C for 10 min, 30 cycles of denaturation at 95 °C for 1 min, annealing at 60 °C for 1 min, extension at 72 °C for 1 min, and the final extension step at 72 °C for 10 min [17]. PCR products were examined by electrophoresis on 1.5% agarose gel at 100V for 45 min; the gel was stained with ethidium bromide and observed under UV light; 100 bp DNA Ladder (Norgen Biotek, Thorold, ON, Canada) was used as a DNA marker. A commercial live attenuate vaccine for *Avipoxvirus* was used as positive control and PCR-grade distilled water as negative control.

### 2.3. Statistical Analyses

The obtained results were analyzed using Chi-square (X^2^) test. Statistical tests were used to compare positive results in relation to species, sex, and provenience (hunting activity or recovery center). The statistical significance threshold was set at a *p*-value ≤ 0.05.

## 3. Results

### 3.1. Samples

Overall, 300 spleen samples were collected. Among them, 196 specimens were collected from the hunted animals: 80 Eurasian teals (*Anas crecca*), 40 common pheasants (*Phasianus colchicus*), 27 mallards (*Anas platyrhynchos*), 24 Eurasian wigeons (*Mareca penelope*), 10 shovelers (*Spatula clypeata*), 4 common shelducks (*Tadorna tadorna*), 4 pintails (*Anas acuta*), 2 Eurasian coots (*Fulica atra*), 2 gadwalls (*Mareca strepera*), 1 tufted duck (*Aythya fuligula*), 1 garganey (*Anas querquedula*), and 1 common pochard (*Aythya ferina*). No bird showed macroscopic lesions reportable to APV infection.

Moreover, spleen samples were collected from 104 birds that died in the recovery center: 45 Eurasian magpies (*Pica pica*), 25 carrion crows (*Corvus corone*), 10 yellow-legged gulls (*Larus michahellis*), 6 common snipes (*Gallinago gallinago*), 5 feral pigeons (*Columba livia*), 4 Eurasian sparrow hawks (*Accipiter nisus*), 3 common kestrels (*Falco tinnunculus*), 2 grey herons (*Ardea cinerea*), 1 peregrine falcon (*Falco peregrinus*), 1 little owl (*Athene noctua*), 1 greylag goose (*Anser anser*) and 1 common starling (*Sturnus vulgaris*). Necroscopies did not reveal gross lesions reportable to cutaneous or diphtheritic forms in any birds. Trauma was recognized as the cause of death for all birds.

Out of the total the studied birds, 112 (37.33%) were males and 188 (62.67%) females.

### 3.2. Molecular Investigations

**Figure 1:**
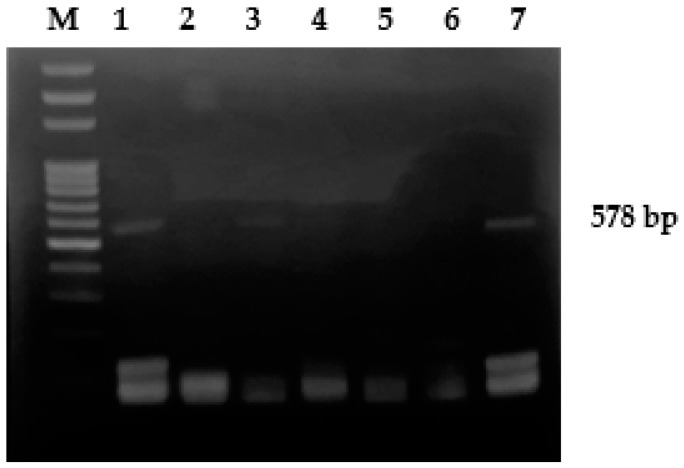
PCR for the amplification of a 578 bp fragment of 4b core protein gene of *Avipoxvirus*. Legend. M: DNA ladder; 1, 3: positive samples; 2, 4, 5: negative samples; 6: negative control; 7: positive control.

Among the 300 tested samples, 130 (43.33%; 95% CI: 37.72–48.94%) were PCR-positive for APV. No amplification was obtained with the negative control, whereas the expected 578 bp fragment was detected with the positive control (Figure 1). However, in order to verify the protocol specificity, four positive samples, randomly chosen (1 from *C. coronae*, 1 from *P. pica*, 1 from *A. noctua*, 1 from *F. atra*) were submitted to sequencing analysis; all of them showed a nucleotide identity of 99% to some *Avipoxvirus* strains.

In detail, APV DNA was detected in 50/112 (44.64%; 95% CI: 35.43–53.85%) males and 80/188 (42.55%; 95% CI: 35.48–49.62%) females.

APV was not found in the samples collected from common pochard (*Aythya ferina*), gadwalls (*Mareca strepera*), Eurasian sparrowhawks (*Accipiter nisus*), peregrine falcon (*Falco peregrinus*), grey herons (*Ardea cinereal*), greylag goose (*Anser anser*), and common starling (*Sturnus vulgaris*).

Considering the avian species for which higher numbers of individuals were tested, relevant percentages of positivity were detected among *L. michahellis* (10/10, 100.00%), *A. platyrhynchos* (17/27, 62.96%; 95% CI: 44.74–81.18%), *S. clypeata* (6/10, 60.00%; 95% CI: 29.64–90.36%), *A. crecca* (39/80, 48.75%; 95% CI: 37.80–59.70%), and *M. penelope* (11/24, 45.83%; 95% CI: 25.90–65.76%). The obtained results in relation to the investigated avian species are summarized in Table 1.

No statistical differences were observed between males and females or between animals from hunting activities and the recovery center (*p* > 0.05). *Anseriformes* were more frequently infected (*p* < 0.05), with a prevalence of 52.87% (95% CI: 45.06–60.68%), than raptors, in which a 22.22% prevalence (95% CI: 0.00–49.38%) was found, and synanthropic/peri-synanthropic birds (33.58%, 95% CI: 25.58–41.58%).

Finally, aquatic birds (water fowls, snipes, gulls, herons), with 97/175 positive samples, showed a higher total percentage of positivity (*p* < 0.05), 55.42% (95% CI: 48.06–62.78%), than the remaining animals, 26.40% (95% CI: 18.67–34.13%).

## 4. Discussion

Avian pox still represents an issue for poultry health, even though vaccinations are usually performed to protect the breeding. Large outbreaks or small disease events involving few animals were often reported worldwide in domestic [8,18], captive [19,20,21], and wild birds [12,22].

The results obtained in the present molecular survey, with a relevant overall prevalence (43.33%) of APV-positive samples, show that APVs are largely circulating among wild birds of different orders and species in Central Italy. All tested birds seemed to be healthy. Necropsies of the animals sent by the recovery center did not evidence typical cutaneous or diphtheritic lesions. For the hunted birds, accurate observations of the skin of heads and legs, as well as of the mucous membranes of oral and nasal cavities, pharynx, and larynx, were carried out and no typical macroscopic lesions were found. Usually, molecular diagnoses are carried out to test cutaneous and/or mucous lesions; considering that, in our study, no animals showed typical lesions, spleens were chosen to search APVs, assuming that this organ is reached by viruses in the viremic phase.

It is well known that avian pox may be the cause of severe effects not only in domestic poultry but also in wild birds. Elevated predation among affected birds, secondary infections, trauma, reduced male mating success, and death have been related to APV infections [23]. Consequently, avian pox has been identified as an important risk factor in the conservation of small and endangered populations, particularly in island bird species [24].

The involvement of wild birds in the epidemiology of APV infections in Italy has been demonstrated by disease events, as previously reported [12,13,14]. To the best of our knowledge, no data about prevalence rates of APVs spreading in healthy avian populations in Italy and worldwide are available; thus, our results are not comparable to those reported in the literature. In fact, most studies focused on virus characterization in birds with proliferative skin lesions mainly of legs, feet, or head. Moreover, the prevalence of APV infection has been often evaluated through the inspection of pox-like lesions, although, in these cases, the diagnosis was presumptive [9].

However, prevalence values of APV infections observed in wild birds in different geographic areas during surveillance investigations based on lesion detection as screening method were usually low, ranging between 0.0% and 13.0%. Rare large outbreak events have been reported, with a prevalence of up to 88.0% [9].

No statical differences were observed, in our study, between male and female birds. This finding is in agreement with some studies that reported inconsistent results regarding sex bias in APV infections [9,25,26].

Relevant percentages of positivity were detected when testing *Anseriformes* and other birds living in humid areas, such as *G. gallinago* and *L. michahellis*. The order *Anseriformes* includes numerous species previously found to be infected by APVs [9]. Water fowls, as well as other aquatic birds, may be frequently exposed to APVs because they live in environments where hematophagous arthropods are abundant. In fact, the most frequent transmission route of this virus is via biting insects, mostly midges (Ceratopogonidae) and mosquitoes (Culicidae) [27,28]. Other arthropods may act as mechanical vectors of the virus: lesser mealworm (*Alphitobius diaperinus*), fleas (*Parapsyllus longicornis*), and blowflies (*Phaenicia* sp.) [29,30,31].

Climatic changes observed in recent years induced increasing circulation of hematophagous arthropods, including mosquitos, in several geographic areas. Some authors observed that APV infection prevalence in wild birds increased after vector population peaks [32,33,34], and it was also lower at high altitudes, where arthropods were less abundant [35,36].

*Anseriformes* are migratory birds able to fly for long distances; thus, they could act as reservoirs and carriers of APV strains from one geographic area to another. Information about APV in *Anseriformes* is scanty, and to the best of our knowledge, only a few old reports described the disease in these birds [37,38,39]. 

During the present survey, relevant percentages of positivity were observed in *Passeriformes* too. Rates of 33.33% in *P. pica* and 40% in *Corvus coronae* confirmed that Passeriformes birds are frequently affected by APVs, also in view of the numerous avian species belonging to this order [9].

## 5. Conclusions

The results obtained in the present investigation suggest that APVs are largely circulating among wild birds of different orders and species in Central Italy. The relevant overall prevalence (43.33%) of APV-positive wild birds suggests a large circulation of APVs in the studied area, although the examined birds did not show typical lesions. On the basis of this finding, it seems that wild birds could be asymptomatic carriers, as also supposed by Ha et al. [40], who detected a very high prevalence (69.2%) for APV infections during a serosurvey in healthy Passeriformes in New Zealand.

*Anseriformes* and other birds living in aquatic/humid environments seem to be more frequently involved in the epidemiology of these viruses, even though the APV DNA was found in spleen samples of other avian species too.

Our survey simply investigated the prevalence rate of APV infections in wild avifauna. Further studies should be performed to verify species, clades, and subclades of the involved viruses in order to better understand the role of asymptomatic birds as possible sources of infection for other wild birds but also for those kept in captivity in farms, rescue centers, zoos, and domestic aviaries.

## Figures and Tables

**Table 1:** Results of PCR for the detection of *Avipoxvirus* in spleen samples in relation to avian species.

	**Common Name**	**Scientific Name**	**Examined**	**Positive**	
				**N**	**%**
Anseriformes	Eurasian teals	*Anas crecca*	80	39	48.75
	Eurasian wigeon	*Mareca penelope*	24	11	45.83
	Common shelduck	*Tadorna tadorna*	4	3	75.00
	Mallard	*Anas platyrhynchos*	27	17	62.96
	Shoveler	*Spatula clypeata*	10	6	60.00
	Common pochard	*Aythya ferina*	1	0	0.00
	Eurasian coot	*Fulica atra*	2	2	100.00
	Tufted duck	*Aythya fuligula*	1	1	100.00
	Gadwall	*Mareca strepera*	2	0	0.00
	Garganey	*Anas querquedula*	1	1	100.00
	Pintail	*Anas acuta*	4	3	75.00
	Greylag goose	*Anser anser*	1	0	0.00
	Total		157	83	52.87
Raptors	Eurasian sparrowhawk	*Accipiter nisus*	4	0	0.00
	Peregrine falcon	*Falco peregrinus*	1	0	0.00
	Little owl	*Athene noctua*	1	1	100.00
	Common kestrel	*Falco tinnunculus*	3	1	33.33
	Total		9	2	22.22
Synanthropic and semi-synanthropic birds	Grey heron	*Ardea cinerea*	2	0	0.00
	Common pheasant	*Phasianus colchicus*	40	4	10.00
	Eurasian magpie	*Pica pica*	45	15	33.33
	Carrion crow	*Corvus corone*	25	10	40.00
	Yellow-legged gull	*Larus michahellis*	10	10	100.00
	Domestic pigeon	*Columba livia*	5	2	40.00
	Common snipe	*Gallinago gallinago*	6	4	66.67
	Common starling	*Sturnus vulgaris*	1	0	0.00
	Total		134	45	33.58
Total			300	130	43.33

## Data Availability

The data presented in this study are available within the article.
